# Management of post-operative pseudomonas aeruginosa infection in head and neck free flap patients: Systematic review and case series

**DOI:** 10.1016/j.jpra.2026.07.009

**Published:** 2026-07-08

**Authors:** Munir Abukhder, A. Chandegra, J. Ismail, A. Farrow, A. Lima Soares, L. Naderi, Karl F. Payne, C. Schilling

**Affiliations:** a University College London Hospital, London, UK; b Luton & Dunstable Hospital, Luton, UK; c King’s College London, London, UK; d Head and Neck Academic Centre, Division of Surgery and Interventional Science, University College, London, UK

**Keywords:** Acetic acid, Pseudomonas aeruginosa, Infection, Head and neck

## Abstract

**Introduction/Aims:**

Pseudomonas aeruginosa (PA) is isolated in up to 12% of post-operative head and neck cancer patients, capable of surgical site tissue invasion triggering acute inflammation, thrombus formation, and localized microcirculatory collapse. This may result in flap failure and delay post-operative radiotherapy due to extended hospital stays. This report reviews literature on acetic acid washouts as adjunctive treatment for post-surgical PA infection and presents a single-centre case series.

**Methods:**

A systematic review protocol was registered with PROSPERO, and a comprehensive literature search on acetic acid use in PA infection was conducted. A retrospective single-centre review of head and neck patients undergoing ablative and reconstructive surgery with microvascular free tissue transfer (2020–2023) was performed. Patients with clinical and biochemical evidence of PA infection received 2–3 daily bedside washouts with 0.05% acetic acid for 7–14 days, alongside intravenous antibiotics.

**Results:**

Six patients with post-operative PA infection were identified. Acetic acid washouts were administered for 1–2 weeks alongside IV antibiotics based on susceptibility testing. Four patients required return to theatre (mean days post-op=20.5; range=5–44). Post-operative inpatient stays ranged from 9 to 38 days. No flap failures or metal-work removals occurred. Our literature search found no comparable studies.

**Conclusions:**

Acetic acid is an easily available, non-toxic, inexpensive topical agent, with bedside washout courses costing £28–£84 per patient. This modest expense is outweighed by the substantial health and psychological benefits of avoiding flap failure. Its low side effect profile supports its use in managing PA-infected surgical sites post-operatively in head and neck cases.

## Introduction

Free tissue transfer (free flap surgery) is the gold standard for reconstructing complex defects following head and neck cancer ablation. Expected flap survival rates exceed 94%, failure within the first 48 hours is usually caused by vascular comprise but after this time infection is the predominant factor.^1^, ^2^, ^3^ Treatment of infection in head and neck cancer surgery patients can be complex due to physiological decompensation from pre-operative malnutrition, presence of foreign bodies (such as titanium fixation plates), prior radiotherapy and difficulty in isolating clinically relevant pathogens due to communication with the oral cavity.

Among the various pathogens involved in post-operative infections, *Pseudomonas aeruginosa* (PA), a gram-negative, aerobic, non-spore forming rod is noteworthy due to its aggressive nature and propensity to cause tissue damage and vascular compromise.^4^ Park et al. reported PA infection in up to 12% of post-operative head and neck cancer patients.^5^ The ability of PA to form a biofilm makes it difficult to eradicate with systemic antibiotics alone.^13^^,^^14^ This is particularly relevant in cases of composite free flap reconstruction supported by non-biological implanted material (titanium plates). In the most radical effort to eradicate PA infection, all non-biological material is removed and replaced only once the infection has been controlled. In order to avoid disturbance of the reconstruction, topical treatment to the infected site (wound irrigation) combined with systemic antibiotics may clear infection without the need for aggressive surgical intervention.

Acetic acid has antimicrobial bacteriostatic properties and the ability to disrupt bacterial biofilms. Compared to 0.9% sodium chloride washouts, acetic acid has been shown to have superior efficacy in eliminating PA but its use has not been widely documented in the post-surgical setting.^6^, ^7^, ^8^, ^9^, ^10^, ^11^ In this report we present a systematic review of the literature of acetic acid eradication of post-operative PA infection as well as presenting a single centre case series.

## Methods

### Search strategy and information sources

A systematic review protocol was registered with PROSPERO (CRD42023437598). This review is reported in accordance with the Preferred Reporting for Items for Systematic Reviews and Meta-Analyses (PRISMA). Comprehensive electronic search strategies were developed for each database using a combination of relevant keywords and index headings. A total of four bibliographic databases were searched (Medline, Embase, CINAHL, Scopus). The search strategy was modified so that the index headings relevant to each specific database were selected. The search strategy was peer-reviewed by an information specialist. Example of search strategies and results are contained in Supplement 1.

### Eligibility criteria

Inclusion criteria were primary research papers including cohort studies, case series and randomised control trials, investigating the efficacy of diluted acetic acid washouts in the treatment of PA infected surgical wounds of head and neck cancer patients that have undergone free flap reconstruction; and articles published or translated into English. Exclusion criteria were articles which did not investigate the efficacy of diluted AA; articles which included patients that did not undergo free flap reconstruction; articles which utilised pedicled flaps in reconstruction; abstracts; and non-English articles. Two independent reviewers screened titles and abstracts according to the aforementioned inclusion and exclusion criteria. The remaining articles were downloaded in full-text format and re-screened. Discussion with a senior author to achieve consensus resolved any conflicts between the two reviewers.

### Case series patient selection

Retrospective single centre review of head and neck (H&N) patients undergoing major ablative and reconstructive surgery with microvascular free tissue transfer between, 2020 and, 2023 was undertaken. Eligible patients had clinical and biochemical evidence of postoperative surgical site (neck) infection and PA isolated from cultured samples. Baseline characteristics collected included age, gender, past-medical history, and clinical staging (AJCC 8th edition). In addition, medical records were analysed to review surgical procedure, time to PA infection diagnosis, number of returns to theatre, antibiotic regimen, number of acetic acid washouts and any noted side effects, confirmed negative culture post washout and length of hospital stay.

### Management protocol

Trust guidelines for antibiotic prophylaxis in this type of surgery recommend a single administration of intravenous (IV) Co-Amoxiclav 1.2 g at induction, followed by two further administrations of the same dose at 8-hour intervals. For patients exhibiting clinical and biochemical signs of infection, empirical antibiotic therapy was initiated promptly and subsequently adjusted based on the identification of PA from cultured swabs and antimicrobial susceptibility testing. The decision to escalate to surgical intervention (return to theatre or image-guided drainage) was based on clinical indicators including suspected abscess formation, failure of infection to respond to initial antibiotic therapy, wound dehiscence, or concern regarding vascular compromise of the flap. Where applicable, additional microbiological samples were obtained during these procedures.

In the absence of a pre-existing protocol and given the limited evidence available at the time, the implementation of AA therapy was carried out in a pragmatic manner. Furthermore, as AA was used in an off-licence context, audit data were collected for submission to the hospital formulary committee. Consequently, the project was registered in accordance with the UCLH audit policy as a service development and clinical audit. In all cases, bedside washouts with 0.05% acetic acid were performed two to three times daily, with treatment duration ranging from 7 to 14 days based on clinical response.

## Results

### Literature search result

No relevant studies were identified following a comprehensive search of four bibliographic databases (Medline, Embase, CINAHL, Scopus).

### Case series

During the study period 223 patients underwent free flap reconstruction. A total of 6 patients met the inclusion criteria for this study (5 males and 1 female) with a mean age of 63.34 years (range = 53 - 68). PA infection rate following free flap surgery in the H&N was thus 2.7%.

All six patients were classified as American Society of Anaesthesiologists (ASA) class III, and four patients had pre-existing diagnosis of non-insulin dependent diabetes mellitus. Free tissue transfer included fibula free flap (FFF) (n=3), anterior lateral thigh (ALT) flap (n=1), radial forearm free flap (RFFF) (n=1) and scapular tip free flap (SFF) (n=1). Table 1 summarises patient demographics, diagnosis, and surgical procedures.

**Table 1:** _Patient demographics, diagnosis, and primary operations._

**Patient number**	**Age**	**Gender**	**Diagnosis**	**Comorbidities**	**ASA**	**Previous radiotherapy**	**Primary operation**
1	62	Female	T4aN3M0 SCC right mandible	T2DM, asthma	3	No	Segmental mandibulectomy, FFF, bilateral modified radical ND
2	53	Male	T4a N0 M0 SCC right mandible	Hypertension	3	No	Segmental mandibulectomy, right selective ND, left FFF
3	64	Male	T4aN1M0 SCC right retromolar trigone	Hypertension, hypothyroidism	3	No	Segmental mandibulectomy, right neck selective ND, and FFF
4	66	Male	T4aN2c SCC tongue	T2DM, hypertension	3	No	Subtotal glossectomy, bilateral ND, right ALT perforator free flap
5	68	Male	T4N0M0 SCC posterior left buccal mucosa	T2DM, hypertension, hypercholestrolaemia, cardiomyopathy (apical hypertrophy)	3	No	Left Segmental Mandibulectomy and SFF
6	67	Male	T3N0M0 SCC right alveolus	T2DM, hypothyroidism, hypertension, hypercholestrolaemia	3	No	Right mandibular rim resection, bilateral ND, and left RFFF

SCC: Squamous Cell Carcinoma FFF: fibula free flap ND: neck dissection ALT: antero-lateral thigh SFF: scapula tip free flap RFFF: radial forearm free flap

The duration of inpatient stay varied considerably among patients, and was often extended due to the complexity of infection and the need for multiple interventions. Post-operative inpatient stays ranged from 9 to 38 days. However, two patients required re-admission for further investigations and management due to PA infection, resulting in additional hospital stays of 11 to 16 days each. First visible signs of PA infection appeared between 2- and 15-days post-surgery. The timing of introducing acetic acid washouts also varied; in some cases, it was initiated within the first two weeks post-operatively, while in others, it followed initial management failures such as a trial with antibiotics alone. The persistence of PA, resistant to initial antibiotics, prompted the use of acetic acid in all cases, which eventually led to clinical improvement in all patients, and negative wound cultures in 4/6 cases. All antibiotic regimens, regardless of whether acetic acid was initiated or not, were discussed with a microbiologist once PA was confirmed on MC&S. Table 2 summarises isolated organisms within operative sites, the selected antibiotic regimen, the incidence of return to theatre, and whether final clearance of PA was achieved.

**Table 2:** _operative site infection information, including organisms grown, return to theatre, and final clearance through culture._

**Patient**	**Routine post-operative** **antibiotic**	**Isolated organisms**	Intravenous Antibiotic therapy given after PA infection confirmed*.	**Return to theatre** **Y/N (day)**	**Confirmed negative swab culture prior to discharge** **(Y/N)**
1	2 doses of co-amoxiclav	P. aeruginosa S. epidermidis K. pneumoniae	1. Piperacillin/tazobactam 2. Teicoplanin 3. Ciprofloxacin	Y (16)	Y
2	2 doses of co-amoxiclav	P. aeruginosa S. epidermidis	1. Piperacillin/tazobactam	Y (44)	Y
3	2 doses of co-amoxiclav	P. aeruginosa F. nucleatum	1. Piperacillin/tazobactam 2. Vancomycin	Y (5)	N
4	2 doses of co-amoxiclav	P. aeruginosa S. constellatus	1. Piperacillin/tazobactam	Y (17)	N
5	2 doses of co-amoxiclav	P. aeruginosa K. aerogenes C. albicans	1. Piperacillin/tazobactam 2. Meropenem	N	Y
6	2 doses of co-amoxiclav	P. aeruginosa	1. Piperacillin/tazobactam 2. Meropenem	N	Y

⁎ Number indicates subsequent courses of antibiotics. Decision to change antibiotics based on lack of clinical improvement or proven drug resistance.

Ultrasound guided drainage of collection within the recipient (flap) site was undertaken in two patients, in both cases the procedure was undertaken more than once. A return to theatre with exploration and application of 5% acetic acid for 10 minutes followed by washout with 0.05% solution was initiated in four patients, and once AA washouts were instituted only one patient required a further visit for washout under general anaesthetic. In this case surgical exploration and washout with 0.05% acetic acid due to a communication between the neck and tracheostomy site was initiated by EUA, followed by a second EUA in theatre a week later. The mean post-operative day to return to theatre was 20.5 days.

Any interventions were followed by a series of bedside acetic acid washouts, lasting from one to two weeks, in a sustained effort to control infection locally. In all cases no debridement of the free flap tissue was required. The successful use of acetic acid washouts in conjunction with systemic antibiotics resulted in patient discharge, with no flap failures or need to explant metalwork.

## Discussion

Post operative infection of the recipient (flap) site is a cause of late flap failure (>48 hours post-operatively).^12^^,^^13^ *Pseudomonas aeruginosa* infection is of notable concern, primarily due to its capacity to thrive in hospital settings and possibility of resistance to multiple antibiotics.^5^^,^^14^

PA is well recognised for its ability to form biofilms, particularly on prosthetic material; however, the presence of mature biofilms was not directly demonstrated in our cohort.^15^ In the early post-operative setting, infections are likely to represent a continuum from planktonic bacterial colonisation to early biofilm formation, rather than established chronic biofilms. Nevertheless, the presence of foreign material in four of our cases may have facilitated early biofilm development. Within the innermost colonies of the biofilm “persister cells” remain more dormant with altered bacterial growth and often with increased antimicrobial resistance which can be implicated in late onset infections. The induction of biofilm specific genes within the colonies of bacteria along with reduced penetration of antibiotic drugs at the infection site reduce the efficacy of systemic antibiotics, decreasing the probability of achieving sufficient antibiotic levels above the minimum inhibitory concentration (MIC) at the site of infection to achieve a therapeutic effect.^16^, ^17^, ^18^

In our series of six patients with post-operative PA infection all were successfully treated with 0.05% acetic acid washouts and intravenous antibiotics, PA infection developed within the first two weeks in 83.34% of cases (n=5). One patient presented with infection at day 40. This is rather unusual, but we theorise that antibiotic treatment during the inpatient stay was sufficient to inhibit but not eradicate the biofilm persister cells. In this case the patient had a fibula free flap supported by a reconstruction plate. The imaging connivingly demonstrated a perimandibular collection and cultures isolated PA.

Four cases had implanted material at the infection site, either in the form of reconstruction plates to support the neo-mandible or miniplates to support the reconstructed maxilla. In these patients infection was controlled with IV antibiotics and 0.05% acetic acid washouts without the need to explant metalwork.

Weak organic acids (such as acetic acid) can interfere with PA colonisation due to perturbation of the biofilm, diffusion of ions across the biofilm membrane through lipophilic properties and accumulation of anions into bacterial cytoplasm leading to osmosis altering intracellular processes.^19^^,^^20^ Numerous studies have demonstrated the successful use of topical acetic acid in eradicating pseudomonas from wound infections.^8^^,^^21^^,^^22^ Sloss et al. reported elimination of PA from burns and soft tissue wounds within two weeks using 0.5–5% acetic acid, and Nagoba et al. demonstrated similar efficacy with 3–5% solutions within 12 days.^9^^,^^23^ In the present series, a lower concentration (0.05%) was selected pragmatically, primarily based on safety considerations in the post-operative head and neck setting, where preservation of flap viability and avoidance of local tissue irritation are paramount. Higher concentrations reported in the literature are largely derived from studies in burns and chronic wounds, which may not be directly applicable to this context. In the absence of established protocols, a more dilute solution was chosen to balance potential antimicrobial efficacy with tolerability. Kundukad et al. utilising acetic acid with pH < pKa (the weak acids undissociated form) demonstrated its ability to penetrate the pseudomonas biofilm matrix resulting in organism death. The ideal pH of the acid should be between 3.3 – 3.5. At this level, the solution is safe to use on human tissue but equally effective at killing pseudomonas.^17^ The efficacy of acetic acid has also been demonstrated to extend to multiple drug-resistant bacteria in both the planktonic and biofilm forms.^6^

The financial cost of bedside acetic acid washouts ranges from £28 to £84 per patient. This expense is outweighed by the substantial health and psychological costs associated with flap failure. Additionally, the low side effect profile of acetic acid further supports its use in this context.

## Limitations

A key limitation of this study is the small sample size, reflecting the relatively low infection rate within our cohort. This limits statistical power and restricts the strength of conclusions that can be drawn regarding treatment efficacy. In addition, all patients received concurrent systemic antibiotic therapy, and several underwent surgical interventions, making it difficult to isolate the independent effect of acetic acid. As such, the findings should be interpreted with caution and considered hypothesis-generating. Prospective studies with larger sample sizes are required to more comprehensively evaluate the effectiveness of acetic acid, including its optimal concentration and its role in comparison with alternative therapeutic approaches.

## Conclusion

Acetic acid is an easily available, non-toxic and inexpensive topical agent. It may represent a feasible adjunct in the management of P. aeruginosa-infected surgical sites post-operatively in the head and neck, potentially mitigating the necessity for extended antibiotic regimens in patients; however, robust conclusions cannot be drawn from this small case series, and larger prospective studies are required.

## Ethical approval

Not required.

## Funding

None.

## CRediT authorship contribution statement

**Munir Abukhder:** Data curation, Formal analysis, Investigation, Visualization, Writing – original draft, Writing – review & editing. **A. Chandegra:** Data curation, Investigation, Resources, Writing – review & editing. **J. Ismail:** Validation, Writing – review & editing. **A. Farrow:** Conceptualization, Methodology, Supervision, Validation, Writing – review & editing. **A. Lima Soares:** Methodology, Validation, Writing – review & editing. **L. Naderi:** Resources, Validation, Writing – review & editing. **Karl F. Payne:** Methodology, Validation, Writing – review & editing. **C. Schilling:** Methodology, Project administration, Supervision, Validation, Writing – original draft, Writing – review & editing.

## Declaration of competing interest

None declared.

## Appendix Supplementary materials

Image, application 1
