# Supplementary material for: Management of post-operative pseudomonas aeruginosa infection in head and neck free flap patients: Systematic review and case series
Source: JPRAS Open. 2026 Jul 8;51:456–62. doi: 10.1016/j.jpra.2026.07.009 (PMC13446188; doi:10.1016/j.jpra.2026.07.009)
Supplement: Supplementary file 1 [file mmc1.pdf]

Supplement 1. Search strategies and results

Database: Embase <1974 to 2023 May 12>
Search Strategy:

1exp "head and neck metastasis"/ or exp metastasis/ or exp cervical lymph node metastasis/ or exp neck metastasis/ (783118)
2((cancer* or carcinoma* or neoplasm* or tumor* or tumour* or metasta*) adj2 (oral or tongue or pharyngeal or pharynx or Mouth or Intra-oral or Intraoral or Hypopharynx or oropharynx or nasopharynx or "Oral cavity" or "oral squamous cell" or buccal or occult or "lymph node" or "head and neck" or neck)).mp. (390040)
3exp pharynx tumor/ (59746)
4exp "head and neck tumor"/ (382865)
5exp tongue tumor/ (13025)
6exp tonsil tumor/ (3342)
7exp "head and neck metastasis"/ or exp neck metastasis/ (3218)
8"acetic acid*".tw. (57760)
9(Acetasol or vasotate or Domeboro or Vosol).mp. (91)
10exp acetic acid/ (66010)
118 or 9 or 10 (98468)
12wound*.mp. [mp=title, abstract, heading word, drug trade name, original title, device manufacturer, drug manufacturer, device trade name, keyword heading word, floating subheading word, candidate term word] (454579)
13infect*.mp. [mp=title, abstract, heading word, drug trade name, original title, device manufacturer, drug manufacturer, device trade name, keyword heading word, floating subheading word, candidate term word] (3473000)
14pseudomona*.mp. [mp=title, abstract, heading word, drug trade name, original title, device manufacturer, drug manufacturer, device trade name, keyword heading word, floating subheading word, candidate term word] (209077)
15exp wound care/ (113176)
16exp Pseudomonas/ (176784)
1714 or 16 (209077)
1812 or 13 or 15 (3801682)
191 or 2 or 3 or 4 or 5 or 6 or 7 (1140167)
2011 and 17 and 18 and 19 (6)
2111 and 17 and 18 (395)
